# Supplementary material for: High-Protein Mulberry Leaves Improve Glucose and Lipid Metabolism via Activation of the PI3K/Akt/PPARα/CPT-1 Pathway
Source: Int J Mol Sci. 2024 Aug 10;25(16):8726. doi: 10.3390/ijms25168726 (PMC11354309; doi:10.3390/ijms25168726)
Supplement: Supplementary file 1 [file ijms-25-08726-s001.zip › supplementary table.pdf]

Table S1. Compositions of High-protein Mulberry leaves and Mulberry leaves based on UHPLC-MS.

High-protein Mulberry leaves	Mulberry leaves
LAURIMINODIPROPIONIC ACID	(E)-parinaric acid
(E)-parinaric acid	LAURIMINODIPROPIONIC ACID
1-Deoxynojirimycin	Choline
Choline	lactide
N-(4-Hydroxy-3-nitrophenyl)-2-[(1-methyl-2,4,6-trioxohexahydro-5-pyrimidinyl)carbonyl]hydrazinecarboxamide	1,2-di-O-methyl-4-[(2R)-2,4-dihydrobutyramido]-4,6-dideoxy-alpha-D-mannopyranoside
Gaboxadol	L(-)-Pipecolinic acid
L-Leucine	2-Pyrrolidinecarboxylic acid
lactide	N-(4-Hydroxy-3-nitrophenyl)-2-[(1-methyl-2,4,6-trioxohexahydro-5-pyrimidinyl)carbonyl]hydrazinecarboxamide
Propyl 1-ethyl-6-methyl-4-(2-nitrophenyl)-2-oxo-1,2,3,4-tetrahydro-5-pyrimidinecarboxylate	Trigonelline HCl
1-[(3-Carboxypropyl)amino]-1-deoxy-beta-D-fructofuranose	4-[5-Hydroxy-6-(2-hydroxy-3-methyl-4-methylenetetrahydro-2-furanyl)-1,3-dioxan-4-yl]-2,6-piperidinedione
1,2-di-O-methyl-4-[(2R)-2,4-dihydrobutyramido]-4,6-dideoxy-alpha-D-mannopyranoside	8-{3-Oxo-2-[(2E)-2-penten-1-yl]-1-cyclopenten-1-yl}octanoic acid
Adenosine	L-Leucine
Phenylacetylene	L-Phenylalanine
H-Ala-pNA	4-(Trifluoroacetyl)benzoic acid
(1R,2S,5S)-N-(4-Amino-1-cyclopropyl-3,4-dioxo-2-butanyl)-3-[N-(isopropylcarbamoyl)-3-methyl-L-valyl]-6,6-dimethyl-3-azabicyclo[3.1.0]hexane-2-carboxamide	Phenylacetylene
Tetralin	Heptanophenone
2-Pyrrolidinecarboxylic acid	7-Hydroxycoumarine
4-Hydroxyisoleucine	Adenosine
9S,13R-12-Oxophytodienoic acid	DL-TYROSINE
Ethynylcytidine	1-carboxy-3-hydroxyadamantane
Cinnamic acid	5-Hydroxymethylfurfural
7-Hydroxycoumarine	Tetralin
MFCD00027233	Betaine
Chlorogenic acid	Chlorogenic acid
4-Acetamidobutyric acid	Diethyl acetamidomalonate

DIETHYL (BOC-AMINO)MALONATE	MFCD00027233
Stearidonoyl glycine	4-Acetamidobutyric acid
α-Propylaminopentiophenone	α-Linolenic acid
N-BOC-hydroxylamine	Isoscopoletin
2-Oxo-3-piperidinecarbohydrazide	6-Hydroxypicolinic acid
Diethyl acetamidomalonate	Cytosine
4-Guanidinobutyric acid	3-(2,6-Dioxocyclohexyl)propanenitrile
N-{[(2R,4S,5R)-5-{[Methyl(phenyl)amino]methyl}-1-azabicyclo[2.2.2]oct-2-yl]methyl}-2-thiophenesulfonamide	6-Shogaol
Trigonelline HCl	Guanosine
trans-3-Indoleacrylic acid	Stearidonoyl glycine
4-[5-Hydroxy-6-(2-hydroxy-3-methyl-4-methylenetetrahydro-2-furanyl)-1,3-dioxan-4-yl]-2,6-piperidinedione	Phenacetin
Heptanophenone	Thymine
α-Linolenic acid	3-(1-hydroxyethyl)-2,3,6,7,8,8a-hexahydropyrrolo[1,2-a]pyrazine-1,4-dione
Adenosine 5'-monophosphate	(1R,2S,5S)-N-(4-Amino-1-cyclopropyl-3,4-dioxo-2-butanyl)-3-[N-(isopropylcarbamoyl)-3-methyl-L-valyl]-6,6-dimethyl-3-azabicyclo[3.1.0]hexane-2-carboxamide
5-Hydroxymethylfurfural	9-Oxo-10(E),12(E)-octadecadienoic acid
Guanosine	19-Norandrostenedione
Nicotinic acid	α-Propylaminopentiophenone
Piperocaine	L-Pyroglutamic acid
Guvacoline	Isopropyl 4-Hydroxybenzoate
Psychosine	Tropine
Cytosine	Pyridoxamine
4-(Trifluoroacetyl)benzoic acid	trans-3-Indoleacrylic acid
1-carboxy-3-hydroxyadamantane	Nicotinamide
3-Hydroxy-2-methylpyridine	2,3,4,9-Tetrahydro-1H-β-carboline-3-carboxylic acid
12-Oxo phytodienoic acid	Cytidine
L(-)-Pipecolinic acid	3,4-Dimethoxy-α-pyrrolidinopentiophenone

9-Oxo-10(E),12(E)-octadecadienoic acid	Prolylleucine
4-Methoxy PV8	12-Oxo phytodienoic acid
Betaine	N6-Me-Adenosine
3-(1-hydroxyethyl)-2,3,6,7,8,8a-hexahydropyrrolo[1,2-a]pyrazine-1,4-dione	Cordycepin
Pyridoxamine	6-Methylquinoline
1,2,3,4-Tetramethyl-1,3-cyclopentadiene	Uracil
3-(2,6-Dioxocyclohexyl)propanenitrile	Adenosine 5'-monophosphate
19-Norandrostenedione	7,8-Dihydroxycoumarin
3-Hydroxy-5-(hydroxymethyl)-2-methylisonicotinaldehyde oxime	Estriol
6-Methyl-2-pyridinemethanol	N-Acetyl-L-tyrosine
Cytidine	4-Guanidinobutyric acid
4-(3,4-Dihydroxyphenyl)-6,7-dihydroxy-2-naphthoic acid	Pyridoxine
1,3-Dimethyl-6-morpholino-1,2,3,4-tetrahydropyrimidine-2,4-dione	Guvacoline
Indole	2'-O-Methyladenosine
L-Pyroglutamic acid	2''-O- β -L-Galactopyranosylorientin
6-Hydroxypicolinic acid	13(S)-HOTrE
13(S)-HOTrE	Indole
Estriol	Camphor
Phthaldialdehyde	3-Hydroxy-5-(hydroxymethyl)-2-methylisonicotinaldehyde oxime
5-[(1,3-Dimethyl-1H-pyrazol-5-yl)amino]-5-oxopentanoic acid	3-Furfuryl 2-pyrrolecarboxylate
Uracil	Pyridoxal
Methyprylon	Kojic acid
3,4-Dimethoxy-α-pyrrolidinopentiophenone	Methyprylon
Kojic acid	Paeonol
Tropine	Paracetamol
3,3'-Dimethoxybenzidine	Anisic aldehyde
8-Hydroxyquinoline	N(6)-OH-Me-Adenosine
N6-Me-Adenosine	Cyclo(leucylprolyl)
Pyridostigmine	Curcumenol
Anisic aldehyde	Scopoletin
Sedanolide	Pyrrole-2-carboxylic acid
N-Acetyl-L-tyrosine	2-Hydroxyphenylalanine
Paracetamol	Psychosine
Cyclo(leucylprolyl)	(8aR,12S,12aR)-12-Hydroxy-4-methyl-4,5,6,7,8,8a,12,12a-octahydro-2H-3-benzoxecine-

	2,9(1H)-dione
Artemisinic acid	1,2,3,4-Tetramethyl-1,3-cyclopentadiene
Prolylleucine	Esculetin
Sorbic acid	4-Amino-3-hydroxybenzoic acid
4,6-Dimethyl-2(1H)-pyrimidinone	Sedanolide
Anacardic acid	Protocatechualdehyde
2-Hydroxyphenylalanine	L-Dopa
1,4:3,6-Dianhydro-2-deoxy-5-O-(ethylcarbamoyl)-2-[(phenylcarbamoyl)amino]-D-glucitol	Esculin
L-Dopa	6-Methyl-2-pyridinemethanol
6-Shogaol	Phthaldialdehyde
Fraxinellone	Curdione
Flurandrenolide	Hypoxanthine
Skimmin	3-Methylhippuric acid
Pyridoxine	3-Hydroxy-3,5,5-trimethyl-4-(3-oxo-1-buten-1-ylidene)cyclohexyl β-D-glucopyranoside
Linoleoyl Ethanolamide	8-Hydroxyquinoline
Phenacetin	Clareolide
1-(4-Methylphenyl)pyrrolidine-2,5-dione	Meperidine
Metaproterenol	Norfenefrine
Meperidine	Sorbic acid
Myriocin	Arecoline
5'-S-Methyl-5'-thioadenosine	1-Deoxynojirimycin
(8aR,12S,12aR)-12-Hydroxy-4-methyl-4,5,6,7,8,8a,12,12a-octahydro-2H-3-benzoxecine-2,9(1H)-dione	4,6-Dimethyl-2(1H)-pyrimidinone
Stachydrine	Cuminaldehyde
Piceatannol	4-Methoxy PV8
Anhydroecgonine methyl ester	Astragalin
Norfenefrine	4-Indolecarbaldehyde
Caffeic acid	Leucylproline
Acetophenone	(-)-Caryophyllene oxide
Salbutamol	Phenmetrazine
Isoscopoletin	Fraxinellone
1,5-Isoquinolinediol	Isoquercitrin
p-Coumaric acid	Isoalantolactone
Ginkgolic acid (C13:0)	Caffeic acid
Esculetin	Betaxolol
SB236057A	Piperocaine
Stearamide	Docosahexaenoic acid methyl ester

Esculin	o-Veratraldehyde
Guanine	Metalaxyl
(1R,2S,3R,4R)-3-[(Cyclopentylmethyl)amino]-4-phenyl-1,2-cyclopentanediol	Epinephrine
2-Hydroxy-4-methoxybenzaldehyde	L-Tyrosine methyl ester
2'-O-Methyladenosine	Cyclo(phenylalanyl-prolyl)
6-Methylquinoline	Coumarin
L-Tyrosine methyl ester	Stearamide
O-Desmethylvenlafaxine	4-Methyl-6,7-dihydroxycoumarin
Butyl 4-aminobenzoate	Orsellinic acid
Palmitoleic acid	Quercetin
Cyclo(phenylalanyl-prolyl)	4-Methoxycinnamaldehyde
Rutin	(9aR,9bS)-9a-Hydroxy-6,9-dimethyl-3-methylene-3,3a,4,5,9a,9b-hexahydroazuleno[4,5-b]furan-2,7-dione
17α-Methyl-androstan-3-hydroxyimine-17β-ol	Isosteviol
Sinapic acid	Pyridostigmine
Orsellinic acid	Linolenic acid ethyl ester
N-Acetyldopamine	Brazilin
o-Veratraldehyde	3-Butylidenephthalide
Thymine	2-Hydroxy-4-methoxybenzaldehyde
F2201	N-Acetyldopamine
Curcumenol	Benzoic acid
PEG n8	2,4-Dimethylbenzaldehyde
7-(2-Chloroethyl)theophylline	Linoleoyl Ethanolamide
Camphor	5-Hydroxy-1-tetralone
Linolenic acid ethyl ester	Anacardic acid
Tapentadol	Fisetin
Cuminaldehyde	7-Methoxycoumarin
1,3-Divinyl-2-imidazolidinone	Abietic Acid
Phenmetrazine	Norharman
3-Furfuryl 2-pyrrolecarboxylate	Myriocin
Clareolide	Sinapic acid
Retrorsine	Kynurenic acid
3-Hydroxy-3,5,5-trimethyl-4-(3-oxo-1-buten-1-ylidene)cyclohexyl β-D-glucopyranoside	3,4-Dimethoxymethcathinone
Laurolactam	p-Coumaric acid
PEG n7	Ethyl 4-(2-methoxyphenyl)piperazine-1-carboxylate

Isoalantolactone	4-(3,4-Dihydroxyphenyl)-6,7-dihydroxy-2-naphthoic acid
3,4-Dimethoxymethcathinone	Metaproterenol
7,8-Dihydroxycoumarin	1,3-Divinyl-2-imidazolidinone
Atenolol	Flurandrenolide
Mepenzolate	Quinoline
N(6)-OH-Me-Adenosine	Anhydroecgonine methyl ester
Scopoletin	Propyl 1-ethyl-6-methyl-4-(2-nitrophenyl)-2-oxo-1,2,3,4-tetrahydro-5-pyrimidinecarboxylate
Haplamine	(+/-)12(13)-DiHOME
Betaxolol	Palmitoleic acid
Salmeterol	Oxyresveratrol
Curdione	Sepiapterin
Ethyl 4-(2-methoxyphenyl)piperazine-1-carboxylate	3,5-Dimethoxy-4-hydroxybenzaldehyde
4-Indolecarbaldehyde	7-Demethylsuberosin
Resveratrol	tert-Butyl N-[1-(aminocarbonyl)-3-methylbutyl]carbamate
7-Methoxycoumarin	Viloxazine
5-Ethylcyclohexane-1,3-dione	Abscisic acid
(-)-Caryophyllene oxide	Decoquinate
Abscisic acid	Atenolol
1-Linoleoyl glycerol	Bis(4-ethylbenzylidene)sorbitol
Viloxazine	TDIQ
2''-O-β-L-Galactopyranosylorientin	Docosahexaenoic acid
Echinocystic acid	2-{2-Oxo-2-[4-(1H-pyrrol-1-yl)piperidino]ethoxy}acetic acid
PEG n6	1,3-Dimethyl-6-morpholino-1,2,3,4-tetrahydropyrimidine-2,4-dione
4-Hydroxy-3-[2-(2-hydroxyphenyl)-2-oxoethyl]-2H-chromen-2-one	4-Hydroxy-3-[2-(2-hydroxyphenyl)-2-oxoethyl]-2H-chromen-2-one
tert-Butyl N-[1-(aminocarbonyl)-3-methylbutyl]carbamate	Morin
Sepiapterin	Loureirin B
Metalaxyl	Resveratrol
Quercetin	Echinocystic acid
Cafestol	3,4-Methylenedioxy PV8
2-Isopropyl-6-methyl-4-pyrimidinol	Pseudoephedrine tert-butyl carbamate

Decoquinate	Cynaroside
(+/-)12(13)-DiHOME	Isochlorogenic acid B
Parthenolide	Cafestol
Pseudoephedrine tert-butyl carbamate	Rutin
Kynurenic acid	Senkyunolide A
Dimethocaine	Xanthoxyline
N-{[(2R,4S,5S)-5-Ethynyl-1-azabicyclo[2.2.2]oct-2-yl]methyl}-2-furamide	Actidione
4-Methyl-6,7-dihydroxycoumarin	Amfepramone
N-Acetylhistamine	Notopterol
Abietic Acid	7-(2-Chloroethyl)theophylline
Isobavachalcone	Salbutamol
Fisetin	SB236057A
Bis(4-ethylbenzylidene)sorbitol	Ethyl 1-(5-isoxazol-5-ylthiophene-2-sulfonyl)piperidine-4-carboxylate
Perillene	1,3-Dicaffeoylquinic acid
Amfepramone	N-{[(2R,4S,5R)-5-{[Methyl(phenyl)amino]methyl}-1-azabicyclo[2.2.2]oct-2-yl]methyl}-2-thiophenesulfonamide
Palmitoyl ethanolamide	Linarin
3,5-Dimethoxy-4-hydroxybenzaldehyde	Parthenolide
7-Demethylsuberosin	Mepenzolate
Oleoyl ethanolamide	Docosapentaenoic acid
α-Linolenoyl ethanolamide	Palmitoyl ethanolamide
Dibutyl Fumarate	Corchorifatty acid F
13(S)-HpOTrE	Dibutyl Fumarate
Corchorifatty acid F	Citric acid
Cryptochlorogenic acid	Gentisic acid 5-O-Î²-glucoside
Citric acid	(Carbamoylcarbamoyl)carbamate
(2E,6E)-8-Anilino-3,7-dimethyl-2,6-octadien-1-yl trihydrogen diphosphate	13(S)-HpOTrE
3-[(3R,5R,6R)-3-Carboxy-3,5,6-trihydroxy-1-cyclohexen-1-yl]benzoic acid	Cryptochlorogenic acid
Sucrose	dCt
dCt	(+/-)9-HpODE
N-{[(2-Methyl-2-propanyl)oxy]carbonyl}-L-alpha-aspartyl-L-alanyl-L-alpha-aspartyl-L-aspartic acid	(15Z)-9,12,13-Trihydroxy-15-octadecenoic acid
(+/-)9-HpODE	4-[4-(Glycylamino)benzyl]-5-isopropyl-1H-pyrazol-3-yl beta-D-glucopyranoside

(2S)-2-({(2S)-2-[(Hydroxy{[(2R,3S,4S)-2,3,4-trihydroxy-5-(8-hydroxy-2,4-dioxo-1,3,4,4a,5,10a-hexahydropyrimido[4,5-b]quinolin-10(2H)-yl)pentyl]oxy}phosphoryl)oxy]propanoyl}amino)pentanedioic acid (non -preferred name)	N-Acetyl-L-phenylalanine
3-(4-Bromophenyl)-N-[5-({4-[(4-ethyl-1-piperazinyl)methyl]-3-(trifluoromethyl)phenyl}carbamoyl)-2-methylphenyl]-1H-pyrazole-5-carboxamide	Sucrose
Hoquizil	3-tert-Butyladipic acid
Caffeic acid 3-glucoside	(2E,6E)-8-Anilino-3,7-dimethyl-2,6-octadien-1-yl trihydrogen diphosphate
Oleic acid alkyne	Caffeic acid 3-glucoside
N-[(1E)-3-[(2E)-2-(4-Hydroxy-3,5-dimethoxybenzylidene)hydrazino]-1-(4-nitrophenyl)-3-oxo-1-propen-2-yl]benzamide	Oleic acid alkyne
(15Z)-9,12,13-Trihydroxy-15-octadecenoic acid	(+/-)9,10-dihydroxy-12Z-octadecenoic acid
2-Methylbenzoic acid	Thymidine
N-Acetyl-L-phenylalanine	Dodecanedioic acid
Dodecanedioic acid	2-(4-Methyl-3-cyclohexen-1-yl)-2-propanyl 6-O-(6-deoxy- α -L-mannopyranosyl)- β -D-glucopyranoside
3'-Adenosine monophosphate (3'-AMP)	Calcium pantothenate
Formononetin	Baicalin
3-tert-Butyladipic acid	1-Caffeoylquinic acid
1-Caffeoylquinic acid	Uridine
(+/-)9,10-dihydroxy-12Z-octadecenoic acid	N-Acetyl-DL-tryptophan
10-HDA	10-HDA
9(Z),11(E)-Conjugated linoleic acid	3-(tert-Butyl)-N-[4-(2,3-dihydroimidazo[2,1-b][1,3]thiazol-6-yl)phenyl]-1-methyl-1H-pyrazole-5-carboxamide
Calcium pantothenate	Shikimic acid
16-Hydroxyhexadecanoic acid	3'-Adenosine monophosphate (3'-AMP)
D(-)-Salicin	N-({(2R,4S,5R)-5-[2-Methyl-6-(2-thienyl)-4-pyrimidinyl]-1-azabicyclo[2.2.2]oct-2-yl}methyl)cyclobutanecarboxamide

Uridine	4-{[(3S)-3-{5-[4-(Dimethylamino)phenyl]-1,3,4-oxadiazol-2-yl}-1-pyrrolidinyl]methyl}benzoic acid
Ferulic Acid	9(Z),11(E)-Conjugated linoleic acid
Δ17-6-keto prostaglandin F1α	16-Hydroxyhexadecanoic acid
3-Methoxy-4-hydroxyphenylglycol glucuronide	Schizotenuin A
Methyl 2-{[2-O-(6-deoxy- α -L-mannopyranosyl)- β -D-glucopyranosyl]oxy}benzoate	Dodecyl sulfate
Isoguanosine	Isoguanosine
2-(4-Methyl-3-cyclohexen-1-yl)-2-propanyl 6-O-(6-deoxy- α -L-mannopyranosyl)- β -D-glucopyranoside	Prostaglandin F1α
Prostaglandin F1α	(3R,5R)-1,3,5-Trihydroxy-4-{[(2E)-3-(4-hydroxy-3-methoxyphenyl)-2-propenoyl]oxy}cyclohexanecarboxylic acid
palmitic acid	{(1R,2R)-2-[(2Z)-5-(Hexopyranosyloxy)-2-penten-1-yl]-3-oxocyclopentyl}acetic acid
Baicalin	3,5-di-tert-Butyl-4-hydroxybenzyl alcohol
4-{[(3S)-3-{5-[4-(Dimethylamino)phenyl]-1,3,4-oxadiazol-2-yl}-1-pyrrolidinyl]methyl}benzoic acid	Androsin
Dodecyl sulfate	Salvianolic acid A
(3R,5R)-1,3,5-Trihydroxy-4-{[(2E)-3-(4-hydroxy-3-methoxyphenyl)-2-propenoyl]oxy}cyclohexanecarboxylic acid	palmitic acid
Lariciresinol 4-O-glucoside	Mulberroside A
3-(tert-Butyl)-N-[4-(2,3-dihydroimidazo[2,1-b][1,3]thiazol-6-yl)phenyl]-1-methyl-1H-pyrazole-5-carboxamide	Dehydrocholic acid
Porphobilinogen	7-Formyl-4,6'-dihydroxy-6-(hydroxymethyl)-2',5',5',8a'-tetramethyl-3',4',4a',5',6',7',8',8a'-octahydro-2'H,3H-spiro[1-benzofuran-2,1'-naphthalen]-7'-yl acetate
N α -{[(2R)-1-(4-Piperidinylcarbonyl)-2-piperazinyl]carbonyl}-D-tryptophanamide	ent-Prostaglandin F2α
(2S,5aS,8aR)-2-[3-(4-Hydroxy-1-piperidinyl)-	Formononetin

3-oxopropyl]-6-(1H-imidazol-2-ylmethyl)-1-methyloctahydropyrrolo[3,2-E][1,4]diazepin-5(2H)-one	
Isochlorogenic acid B	19(R)-Hydroxy prostaglandin F1α
D-Raffinose	(R)-3-Hydroxy myristic acid
3,5-di-tert-Butyl-4-hydroxybenzyl alcohol	Salidroside
Manninotriose	Lariciresinol 4-O-glucoside
12-Hydroxydodecanoic acid	Xanthosine
Androsin	1,9b-Dihydroxy-6,6,9a-trimethyl-5,5a,6,7,8,9,9a,9b-octahydronaphtho[1,2-c]furan-3(1H)-one
N-Acetyl-DL-tryptophan	3-[2-(β-D-Glucopyranosyloxy)-4-methoxyphenyl]propanoic acid
Aflatoxin G1	Sulochrin
Danshensu	Pinoresinol 4-O-glucoside
Benzyl cinnamate	Quercetin-3-O- β -D-glucose-7-O-β-D-gentiobioside
S-Adenosylhomocysteine	1-(3,5-Dihydroxyphenyl)-12-hydroxy-2-tridecanyl acetate
5-[(1S,2R,4aR)-5-(Hydroxymethyl)-1,2,4a-trimethyl-1,2,3,4,4a,7,8,8a-octahydro-1-naphthalenyl]-3-methylpentanoic acid	2,5-di-tert-Butylhydroquinone
Pinoresinol 4-O-glucoside	N-[(1E)-3-[(2E)-2-(4-Hydroxy-3,5-dimethoxybenzylidene)hydrazino]-1-(4-nitrophenyl)-3-oxo-1-propen-2-yl]benzamide
Dehydrocholic acid	Ethyl ferulate
N-({(2R,4S,5R)-5-[2-Methyl-6-(2-thienyl)-4-pyrimidinyl]-1-azabicyclo[2.2.2]oct-2-yl}methyl)cyclobutanecarboxamide	Tetradecanedioic acid
1,9b-Dihydroxy-6,6,9a-trimethyl-5,5a,6,7,8,9,9a,9b-octahydronaphtho[1,2-c]furan-3(1H)-one	D-Raffinose
Xanthosine	5-[(1S,2R,4aR)-5-(Hydroxymethyl)-1,2,4a-trimethyl-1,2,3,4,4a,7,8,8a-octahydro-1-naphthalenyl]-3-methylpentanoic acid
Thymidine	Plantamajoside
Calceolarioside B	12-Hydroxydodecanoic acid
11-Dehydro thromboxane B2	Isomucronulatol 7-O-glucoside
Loureirin A	Calceolarioside B

Pterostilbene	(2S,5aS,8aR)-2-[3-(4-Hydroxy-1-piperidinyl)-3-oxopropyl]-6-(1H-imidazol-2-ylmethyl)-1-methyloctahydropyrrolo[3,2-E][1,4]diazepin-5(2H)-one
Coumafuryl	11-Dehydro thromboxane B2
1-(3,5-Dihydroxyphenyl)-12-hydroxy-2-tridecanyl acetate	Oleic acid
3-[2-(β-D-Glucopyranosyloxy)-4-methoxyphenyl]propanoic acid	D(-)-Salicin
Tetradecanedioic acid	(1S,4aR,5S)-5-[(3E)-5-Methoxy-3-methyl-5-oxo-3-penten-1-yl]-1,4a-dimethyl-6-methylenedecahydro-1-naphthalenecarboxylic acid
(1S,4aR,5S)-5-[(3E)-5-Methoxy-3-methyl-5-oxo-3-penten-1-yl]-1,4a-dimethyl-6-methylenedecahydro-1-naphthalenecarboxylic acid	Aflatoxin G1
Oleic acid	Gibberellic acid
Quercetin-3-O-β-D-glucose-7-O-β-D-gentiobioside	13,14-dihydro Prostaglandin F1α
Octyl gallate	Asiatic acid
2,2'-Methylenebis(4-methyl-6-tert-butylphenol)	2,2'-Methylenebis(4-methyl-6-tert-butylphenol)
Hexadecanedioic acid	Nα-{[(2R)-1-(4-Piperidinylcarbonyl)-2-piperazinyl]carbonyl}-D-tryptophanamide
β-Muricholic acid	Kaempferol-3-O-rutinoside
{(1R,2R)-2-[(2Z)-5-(Hexopyranosyloxy)-2-penten-1-yl]-3-oxocyclopentyl}acetic acid	CP 47,497-C8-Homolog C-8-hydroxy metabolite
Trolox	
Salidroside	
(R)-3-Hydroxy myristic acid	
13,14-dihydro-15-keto-tetranor Prostaglandin F1β	
cis,cis-Muconic acid	
Schizotenuin A	
Isofraxidin	

Table S2. Differential targets between High-protein Mulberry leaves and Mulberry leaves.

High-protein Mulberry leaves	Mulberry leaves
CRHR1	CACNA2D1
POLB	SIRT3
DDO	SIRT2
TNKS2	DAO
TNKS	CHRM3
BRD9	CHRM2
DYRK1A	CA13
GLI2	ADRB1
GLI1	ADRB2
MAP3K11	SLC22A12
MAP3K9	PTPRS
CNR1	MPG
CES2	DAPK1
HCAR2	CYP3A4
SLC16A1	CYP2C9
CTSK	CYP2C19
NAAA	CYP1A2
SAE1 UBA2	TRPM8
KDM5A	ESRRA
JAK3	ADRA1B
DRD5	CNR2
NOS3	GPR35
IKBKB	SLC7A5
PDE8B	GABBR2 GABBR1
BCL2A1	KIF11
PHLPP2	FABP1
TRAP1	SCD
PTK2	AXL
CAMK2D	NEK6
LTA4H	NEK2
PDE10A	PIK3R1
PRKCE	CXCR1
PRKCQ	APEX1
DPP8	TOP2A
HTR1F	CAMK2B
HTR5A	AKR1C4
GHSR	AKR1C2
SLC6A9	AKR1C1
PKN2	AKR1A1
FGFR1	SLC6A4
CTSC	CYP24A1

CACNA1S	CCR8
GRM5	CCR5
HNMT	SLC6A3
XIAP	CDK6
BIRC2	ARG1
IRAK4	VDR
CDA	ELANE
OGA	CD22
FUCA1	STS
TK1	CYP27A1
PDE4B	NFE2L2
UBLCP1	TACR1
PTPN2	CYP2A6
PTPN9	CISD1
RPA1	ADRA1A
SLC22A6	ALPG
UGT2B7	ALDH1A1
KDM5C	F2
GABRA2 GABRB2 GABRG2	CFTR
PTGER2	PEPD
HAO1	TDP2
ADORA2B	TYMP
MELK	SAE1
SMO	TGM2
F2R	PARP2
MAPK3	SLC15A1
UBA6	RORA
ROS1	KMT5A
FKBP1A MTOR	NPC1L1
TNK2	CPA3
TOP1	GABRR1
HIF1A	EGLN1
CCR1	CSNK1A1
ITK	CPB2
MMP2	NAT1
PLCG1	ANPEP
GRIN2A GRIN1	ENPEP
GPR52	HTR3A
KNG1	PTPN1
TKT	GPR119
MTNR1A	CAPN1
MTNR1B	TAAR1
CCNT1	OPRM1

CACNA2D2	SRC
MC5R	JAK1
PRF1	TGFB1
TAB1 MAP3K7	SLC18A2
MAP3K7	KIT
IMPDH2	SCN10A
PDPK1	TRPC6
KCNA3	TRPC3
ALK	HTR7
CTSL	HTR2A
ADRA2A	HTR1D
ADRA2C	HTR1A
ADRA2B	AURKB
ADRA1D	PDE1A
HTR6	NOS1
HTR1B	MAP3K8
CDK1	MKNK2
VEGFA	MAPK11
FGF1	CSF1R
HPSE	LRRK2
GLRA1	KHK
GLRA2	OPRK1
PPM1A	JAK3 JAK1
LGALS4	TYK2 JAK2
LGALS8	TYK2 JAK1
FGF2	TYK2 JAK2 JAK1
PTAFR	INSR
MGAM	HRH1
FDFT1	GRK2
SERPINE1	GABRB3 GABRA3 GABRG2
MMP9	GABRA2 GABRB3 GABRG2
MMP1	GABRB3 GABRG2 GABRA1
TYR	CDK8
FUT7	CCNC CDK8
ESR2	CDC7
FYN	BDKRB2
AKR1C4	GRK3
CDK2	PLG
XDH	ALDH2
MPI	AKR1C3
ERN1	TSPO
MMP3	F10
MMP8	SHBG

GPR84	RXRG
NOX4	P2RY12
GSK3A	PTPN11
BMP1	PRKCH
ALOX5AP	OXTR
HRH3	NOS2
CHRNA5 CHRNB2 CHRNA4	GYS1
CYP51A1	MAPK14
DRD4	ACP1
UTS2R	ITGAV ITGB3
TRPV3	HMGCR
MAP2K1	NR3C1
HSD11B2	FFAR2
NR1H3	EDNRB
ACACB	PLA2G4A
CYP1B1	CYP51A1
TUBB3	CCKAR
HMGCR	CD81
RPS6KA1	CSNK2A2
CSNK1D	CSNK2A1
PRKACB	CPT1B
MAP2K2	CPT1A
SLK	SOAT2
FRK	SOAT1
STK36	TYMS
GAK	ALB
EPHA6	SELL
TNIK	IGFBP3
PTK6	F7
MAPK12	TUBB1
RPS6KA6	TLR9
CSNK1E	TAS2R31
RIPK2	WEE1
CDC42BPB	MTOR
DDR2	CHEK1
ACVR1B	ROCK2
DDR1	PIK3CD
NLK	PIP4K2C
ACVR2B	PDF
CSNK1A1L	CHRNA7
CIT	MMP25
CDC42BPG	MAPKAPK2
COQ8B	MIF

LATS2	HTT
STK32B	HDAC9
DCTPP1	HDAC8
QPCT	NCOR2 HDAC3
KMT2A	HDAC3
DOT1L	HDAC11
SUV39H1	HDAC10
INMT	HDAC1
SMYD2	GSTP1
EZH2	HSD17B3
EZH1	DYRK2
SETDB1	CLK1
CARM1	CLK3
PRMT1	OPRD1
EHMT1	CCND1 CDK4
EHMT2	BMP4
DNMT3B	ADAM10
AMD1	HPGD
SRM	SLC2A1
SMS	GART
PRMT5	ADORA2A
NNMT	ADORA1
MTAP	PLA2G2D
MARS	
SETD7	
HAGH	
GRIN2B	
GRIN1 GRIN2B	
EBP	
ADRA1A	
IL2	
TNF	
HK2	
HK1	
GRK1	
CMA1	
CYP26A1	
PLA2G2A	
ACE	
PTGDR2	
RXRA	
CCKBR	
MMP14	

TBXAS1	
ITGAL ICAM1 ITGB2	
PYGL	
PRKAG1 PRKAB1 PRKAA2	
TRPM8	
ITGB1 ITGA4	
MDM2	
PTGIR	
PLA2G1B	
OXER1	
PLEC	
PDE4D	
CYP26B1	
ITGB7 ITGA4	
ADAMTS5	
NR1H2	
PTPRF	
HNF4A	
MME	
NTSR1	
KEAP1	
SLC10A2	
GRM2	
PDE5A	
RXRB	
RBP4	
PIN1	
GCG	
ENPP2	

Table S3. The common elements among "T2DM", "Obesity" and the common and differential targets of High-protein Mulberry leaves and Mulberry leaves.

196 common elements in "T2DM", "Obesity" and "HPM":	132 common elements in "T2DM", "Obesity" and "MLE":	215 common elements in "T2DM", "Obesity" and "Common":
HNF4A	INSR	GSTM1
PLEC	SLC2A1	RNASEH1
ACE	PTPN1	GSR
TNF	ALB	QDPR
VEGFA	TGFB1	CCNE1
FGFR1	VDR	CDK2
IL2	PTPN11	ABAT
MMP2	MTOR	NFKB1
SERPINE1	NFE2L2	TH
MTNR1B	NOS2	DNMT3A
MMP9	ADRB2	DNMT3L
GCG	EDNRB	PCNA
NOS3	CCR5	PRKCZ
FGF2	PIK3R1	PBRM1
XDH	CYP3A4	SMARCA4
CACNA1S	F2	PDE3A
HIF1A	CFTR	PDE3B
TYR	SRC	GRIA1
PRF1	MAPK14	VCP
HSD11B2	PLG	MMP16
PTPN2	CPT1A	SLC9A1
MAPK3	ALDH2	EZR
XIAP	IGFBP3	EIF2AK2
MMP1	CYP1A2	FABP1
MME	CYP2C9	NR1I2
CDK2	HMGCR	NFKBIA
EZH2	SHBG	CASP9
RBP4	NR3C1	LGMN
KNG1	LRRK2	TPSAB1
MAP2K1	CPT1B	CPB1
MMP14	CYP2C19	HDAC4
GLI2	ACP1	CTRC
HMGCR	KIT	KLK3
ESR2	BMP4	ACE2
CNR1	PLA2G4A	ITGB1
MMP3	TRPC6	ADAM12
IKBKB	NOS1	PPIA
NR1H2	HTR2A	APLNR

BMP1	GSTP1	LPAR2
PDE4D	JAK1	CTRB1
PTPRF	ADRB1	ATIC
ADRA2A	OXTR	NR1I3
HK1	PIK3CD	GPER1
HK2	MIF	BIRC3
GRIN2B	CNR2	SI
JAK3	ELANE	ACKR3
PTK2	OPRM1	SPHK2
MAP2K2	SELL	SPHK1
EBP	SOAT1	MAP4K4
MPI	GYS1	CHKA
TKT	F7	PLD2
MGAM	TOP2A	USP14
ALK	HDAC9	ERG
CDK1	SIRT3	HCN4
GLI1	SIRT2	HCN1
SLC10A2	SLC6A4	KDM4B
KDM5C	MAPKAPK2	CCND1
FGF1	CXCR1	CDK4
MAP3K7	CYP2A6	CCND2
PRKCQ	TLR9	CCND3
MMP8	SCD	MKNK1
PDE5A	HTT	FPR1
RXRA	HDAC1	FPR2
PDPK1	FABP1	SUMO1
PRKACB	ARG1	PSEN2
GRK1	F10	EIF2AK3
ACACB	CDK6	HTR2A
NOX4	SOAT2	EIF4H
BIRC2	CCKAR	GABRB3
KMT2A	TYMS	AOC3
ADRA2B	HTR1A	GLP1R
CYP1B1	CSF1R	PAOX
EHMT2	BDKRB2	HDAC5
ENPP2	ADORA1	LAP3
KEAP1	KIF11	F2RL3
IRAK4	NEK2	ACACA
F2R	FFAR2	AURKB
TOP1	TSPO	INCENP
CCKBR	CHEK1	NLRP3
CTSK	PARP2	SCARB1
IMPDH2	GPR35	PTGS2

GHSR	APEX1	MAST3
NR1H3	EGLN1	MDM4
SMO	SLC6A3	TP53
CTSL	SLC7A5	PTGES2
RIPK2	AKR1C3	DAGLA
UTS2R	HDAC8	AGTR2
SLC16A1	ESRRA	EIF4E
DRD4	CAMK2B	KDM3A
ALOX5AP	ANPEP	FTO
DNMT3B	PRKCH	TPMT
ERN1	GPR119	NGFR
PTGDR2	HTR3A	CALCRL
KCNA3	ADORA2A	NOD1
ADRA2C	CD81	GNRHR
CCR1	HPGD	SLC2A1
HPSE	AXL	SLC2A3
PDE8B	PEPD	NMBR
CAMK2D	HDAC3	EPHA2
CRHR1	TACR1	EPHB3
DDR1	ADAM10	MAN1B1
BCL2A1	NAT1	ZAP70
PRMT1	RORA	LYN
PDE10A	CISD1	PIM3
EHMT1	HRH1	AGTR1
DYRK1A	DAPK1	S1PR2
CDC42BPB	STS	CYSLTR1
COQ8B	AKR1C2	SGK1
GSK3A	AURKB	LNPEP
CCNT1	NPC1L1	PER2
TBXAS1	CD22	MAPK14
FDFT1	MMP25	ABCC9
OGA	ADRA1B	KCNQ1
HAO1	WEE1	BRS3
PKN2	MAPK11	PPOX
HCAR2	MKNK2	RASGRP1
CTSC	RXRG	DUT
PTAFR	DYRK2	GRK7
MTAP	ADRA1A	PRPF4
KDM5A	CCR8	MAP3K13
MAPK12	CDK8	MAST1
NTSR1	CPB2	HUNK
CARM1	DAO	FLT1
MTNR1A	ENPEP	DSTYK

CDA	HDAC10	PIK3R1
ADRA1A	CLK3	PIK3CA
PHLPP2	CDC7	ACLY
TRAP1	SAE1	FOLH1
DRD5	KMT5A	SLC37A4
SMYD2	KHK	KDM4D
PTGIR	HDAC11	PRKCD
CDC42BPG	CLK1	NEU2
HTR6		EDNRA
PRMT5		RARB
ADORA2B		RARA
PTK6		CTNNB1
ADRA1D		LIPC
LTA4H		LIPG
OXER1		GIPR
TNKS		ITGAV
HNMT		SSTR5
SUV39H1		CBR1
DPP8		PON1
NLK		MGMT
MAP3K11		DGAT1
HAGH		PCSK7
SRM		IL1B
HRH3		PTGIS
MC5R		MAP2K7
UBA6		JUN
SLK		F2RL1
SETD7		NTRK2
GAK		FFAR4
AMD1		IL6
TNIK		GLUL
NNMT		MB
FRK		CASP3
QPCT		JAK2
DCTPP1		TYK2
STK32B		CHUK
GPR84		RET
UBLCP1		MPO
ACVR1B		PKN1
ACVR2B		NR3C2
CSNK1A1L		RPS6KB1
CIT		KDM1A
LATS2		GABRA5

DOT1L		EP300
INMT		ELAVL1
EZH1		PTPRC
SETDB1		PREP
SMS		SERPINA6
MARS		G6PD
GRIN1		PTGFR
CMA1		GCK
CYP26A1		AKR1B1
PLA2G2A		NOS2
ICAM1		F3
ITGB2		HTR2B
ITGAL		DRD1
PYGL		HTR2C
PRKAB1		STAT3
PRKAA2		AKR1C2
PRKAG1		ALPL
TRPM8		TBXA2R
ITGA4		MMP7
ITGB1		EPHB4
MDM2		SLC6A3
PLA2G1B		FASN
CYP26B1		PFKFB3
ITGB7		AHR
ADAMTS5		RELA
GRM2		MAPT
RXRB		CYP1A1
PIN1		CYP11B1
POLB		CYP11B2
		CYP17A1
		RAF1
		MAPK10
		BRAF
		PRMT3
		IDO1
		GLO1
		ADRB3
		CPT1A
		ABCC8
		CASP1
		CTSA
		MMEL1
		CPT2

		EGLN3
		NR1H2
		NTSR1
		GCG
		ENPP2
